# Phytolacca in Mastitis

**Published:** 1874-09

**Authors:** L. Alexander

**Affiliations:** Yorkville, S. C.


### PHYTOLACCA IN MASTITIS.

By L. ALEXANDER, M.D., of Yorkville, S. C.

*Editor Atlanta Medical and Surgical Journal:*

As the advancement of our profession is, in a measure, due to the interchange of views, through the medium of medical journals, and feeling it the duty of every practitioner to place in the hands of his brother chip (after due trial) all new remedies, I herewith give a brief statement in regard to phytolacca decandra, believing that in it we have a most potent remedy in all mammary inflammations.

Case 1.—Mrs. F., whilst nursing her sixth child, suffered for several days with pain, more or less severe, in both breasts. Thinking that all would probably end well under the use of domestic remedies, medical advice was not solicited until the morning of the 8th day. Having been warned by a severe chill the previous night, she concluded that a physician should be summoned. Upon my arrival I found the patient suffering with high fever. The mammary glands were much distended, and bowels constipated. She was suffering great pain and mental anxiety. She was thoroughly convinced that no means could avert the impending trouble, and insisted that poultices should be applied to encourage pointing. I prescribed a saline aperient, followed by the fluid extract of phytolacca decandra, in fifteen-drop doses, every three hours. At my next visit, the following day, I found the glands soft throughout; no pain, and the mind greatly relieved in regard to the matter.

Called again on the 14th. Found same condition as at first. Continued prescription as before, with like result. On the 21st, while on a visit to her mother, the same difficulty arose for the third time, and as it was not convenient to procure the above medicine, our old remedies were brought into play, which, after a few days of fruitless efforts to abate the suppurative process, compelled a resort to the lancet.

Case 2.—Mrs. C., now nursing her fourth child. With each of her first three children all efforts to allay inflammation of the breast proved useless. The lancet was consequently used, leaving her health much impaired. With this last child, immediately upon the appearance of hardened nodules in the breast, phytolacca decandra was prescribed, as in Mrs. F.'s case, with entire relief in about thirty-six hours. She now keeps the medicine in the house, and fears no further trouble from that source.

In each of the above cases the quantity of milk was greatly lessened by the remedy.

